# Sequential Prefrontal and Temporoparietal Repetitive Transcranial Magnetic Stimulation (rTMS) for Treatment of Tinnitus With and Without Comorbid Depression: A Case Series and Systematic Review

**DOI:** 10.3389/fneur.2022.831832

**Published:** 2022-05-19

**Authors:** Katharine G. Marder, Janice Cho, Ruth Chincanchan, Andrew C. Wilson, Juliana Corlier, David E. Krantz, Nathaniel D. Ginder, Jonathan C. Lee, Scott A. Wilke, Reza Tadayonnejad, Jennifer Levitt, Akira Ishiyama, Michael K. Leuchter, Andrew F. Leuchter

**Affiliations:** ^1^TMS Clinical and Research Service, Neuromodulation Division, Semel Institute for Neuroscience and Human Behavior at UCLA, Los Angeles, CA, United States; ^2^Department of Psychiatry and Biobehavioral Sciences, David Geffen School of Medicine at UCLA, Los Angeles, CA, United States; ^3^Division of Humanities and Social Sciences, California Institute of Technology, Pasadena, CA, USA; ^4^Head and Neck Surgery, David Geffen School of Medicine, University of California, Los Angeles, Los Angeles, CA, United States

**Keywords:** transcranial magnetic stimulation (TMS), tinnitus, major depressive disorder (MDD), treatment, theta-burst stimulation, dorsolateral prefrontal cortex, Heschel's gyrus

## Abstract

**Background:**

Tinnitus distress is related to both the loudness and intrusiveness of the tinnitus percept. Treatment approaches targeting both attentional/limbic and auditory systems may better alleviate tinnitus distress than approaches targeting the auditory system alone.

**Materials and Methods:**

Ten subjects with chronic tinnitus received sequential rTMS treatment involving: 1) excitatory stimulation administered to the left dorsolateral prefrontal cortex (DLPFC) or inhibitory stimulation administered to the right DLPFC, followed by 2) inhibitory stimulation administered to primary auditory cortex (Heschel's gyrus or HG). A systematic literature review was performed to evaluate the existing literature on sequential repetitive Transcranial Magnetic Stimulation (rTMS) treatment approaches for tinnitus. Results of the case series are interpreted in the context of tinnitus neurobiology and the extant literature.

**Results:**

Subjects experienced a significant decrease (average 21.7%) in symptoms on the Tinnitus Functional Index (TFI). Those with tinnitus alone experienced a greater mean symptom reduction than those with comorbid MDD (27.7 vs. 17.0%, respectively). Adverse effects were transient and minor. Literature review confirmed that sequential approaches had some advantages compared to single site rTMS; in general, the addition of 1 Hz treatment at DLPFC was superior to single site rTMS in the short term (1–12 weeks), while the addition of 20 Hz treatment at DLPFC appeared superior in the long term (90–180 days).

**Conclusions:**

Sequential rTMS approaches for the treatment of tinnitus—particularly those administering low-frequency treatment at left DLPFC—merit further investigation.

## Introduction

### Tinnitus Overview and Burden

Tinnitus is defined as the perception of an external auditory stimulus in the absence of an external source (1–3). In cases of chronic or persistent tinnitus, these symptoms have been present for at least 3 or 6 months, respectively (2, 4). Tinnitus is highly prevalent in the United States, with estimates ranging from 8–25% (2, 4–6), and it can significantly impair daily function. Approximately 49% of those with tinnitus will discuss it with a physician, and 20% will require clinical intervention (2, 4, 6). Tinnitus commonly impairs sleep, concentration, cognition, and may eventually result in mood, anxiety, or substance use disorders (2–4, 6, 7). Questionnaires such as the Tinnitus Handicap Inventory (THI, developed in 1996) and Tinnitus Functional Index (TFI, developed in 2012) are used to formally assess disease burden (8–12).

### Tinnitus Mechanisms

The pathogenesis of tinnitus is not well-understood, though neuroimaging studies in both animal models and humans have implicated tinnitus-related hyperactivity in the auditory cortex (13–15). Further work demonstrated that activity changes in non-auditory networks may also be implicated in the pathogenesis of tinnitus (13, 16–23). These changes involve the insula, anterior cingulate cortex, and dorsolateral prefrontal cortex (DLPFC) (13, 15, 20, 21, 23). Accordingly, tinnitus may represent a pleomorphic disorder that arises from aberrant dynamics in several different functional networks (12, 16, 24, 25). As an emotional and cognitive integrator implicated both in the experience of subjective distress and in auditory processing, the DLPFC is proposed as a possible point of intervention that engages multiple different networks (22, 23, 26–28).

### Rationale for rTMS and Multi-Site Treatment

Repetitive transcranial magnetic stimulation (rTMS) is a non-invasive method of brain stimulation that alters the activity of specific brain circuits by repeatedly applying electromagnetic stimulation to targeted brain regions. Different stimulation patterns have differential effects on cortical activity. Inhibitory stimulation patterns include low-frequency (e.g., 1 Hz) rTMS or continuous theta-burst stimulation (cTBS—a type of rTMS utilizing 50 Hz triplet bursts superimposed on a 5 Hz carrier wave). Excitatory stimulation patterns include high-frequency (e.g., 10 or 20 Hz) rTMS and intermittent theta-burst stimulation (iTBS—theta burst rTMS delivered in two-second trains with an eight-second intertrain interval). Low-frequency rTMS and cTBS modalities inhibit cortical activity in targeted and connected regions, while high-frequency rTMS and iTBS modalities enhance cortical activity in targeted and connected regions (29).

Observations of hyperactivity in auditory cortical networks inspired the application of inhibitory rTMS to the auditory cortex as a potential treatment for chronic tinnitus beginning in 2003 (30). After several neuroimaging studies demonstrated aberrant activity in both auditory and limbic networks, researchers began employing multi-site rTMS approaches targeting both the prefrontal and auditory cortices in an attempt to reduce both tinnitus loudness and tinnitus distress (7, 16, 17, 21–23, 27, 28, 30–43). Studies of both single site and sequential approaches for the treatment of tinnitus have mixed results to date (44).

We report here on the safety, tolerability, and efficacy of a sequential limbic-auditory rTMS treatment approach in 10 chronic tinnitus patients with and without comorbid depression. We performed a systematic literature review of sequential rTMS approaches for tinnitus. We contextualize the findings of our case series within the extant literature in this area and make recommendations for future trials of sequential rTMS protocols for tinnitus.

## Methods

### Overview and Subjects

An observational case series examined changes in tinnitus following rTMS treatment at both limbic and auditory network targets. We performed rTMS treatment data collection naturalistically without any experimental manipulations. All subjects (*n* = 10) were treated by the University of California, Los Angeles (UCLA) TMS Clinical and Research Service between August 2016 and August 2019. We provided all participants with written informed consent in this IRB-approved observational study. Subjects were treated in accordance with the 2013 Declaration of Helsinki.

Participants met inclusion criteria if they had TFI scores documented at baseline, completed ten sequential rTMS treatment sessions targeting both HG and DLPFC, and had repeat TFI scores documented following treatment. Table 1 shows demographic data of the sample. We divided the participants into two groups: tinnitus subjects without depression (*n* = 4) and those with co-morbid depression (*n* = 6). One of the tinnitus subjects with depression did not have a TFI score documented after the fifth session and was excluded from the session five analysis. Another subject from the same group did not have a session ten score and was excluded from the session ten analysis.

**Table 1:** Demographics, baseline characterstics, and basic treatment parameters of subjects in each group.

	**Tinnitus and depression**	**Tinnitus only**	***p*** **value**
	***n =*** ** 6**	***n =*** ** 4**	
Female subjects (n)	2	1	0.78
Mean age (+- SD)	58.3 (5.9)	67 (10.2)	0.12
Baseline TFI score (+ - SD)	160.5 (55.7)	156 (74.6)	0.92
Number burst stimulation	2	4	0.08
Number magstim	3	1	0.07
Number magventure	0	3	0.07
Number neurostar	3	0	0.07

### rTMS Treatment

Treatments were administered with either the Neuronetics Neurostar treatment system (Neuronetics, Malvern, PA, USA), the MagVenture MagPro R30 TMS System (MagVenture, Inc.), or the Magstim Rapid2 Therapy System (Magstim, Whitland, South Wales, UK). Motor threshold (MT), the minimum stimulus intensity necessary to elicit a motor response in the right abductor pollicis brevis (APB) or first dorsal interosseus (FDI) muscles for 50% of applied stimuli, was determined prior to the first treatment. Following MT measurement, patients were seated in a semi-reclined position using standard safety procedures and ear protection before treatments were administered. The stimulating magnet was placed over the left or right DLPFC using the Beam F3 method and over the HG target using the method described by Langguth et al. (45, 46).

All participants received five consecutive rTMS treatments per week. Each treatment consisted of DLPFC stimulation (either excitatory left DLPFC stimulation or inhibitory right DLPFC stimulation) followed by inhibitory stimulation targeting HG. Inhibitory stimulation involved either 1,000 pulses at 1 Hz (tonic-type stimulation) or 600 pulses of cTBS. Excitatory stimulation involved either 3,000 pulses at 10 Hz (40-pulse train for 4 seconds, intertrain interval of 26 seconds, total duration 37.5 min, tonic-type stimulation) or 600 pulses of iTBS. Treatment was initiated at 80 to 90% MT and advanced as tolerated to a goal intensity of 100 to 120% MT for most patients. Stimulation frequencies, intensities, and pulse numbers per session could be adjusted where needed based on clinician discretion. Two subjects received inhibitory stimulation to bilateral HG from the beginning of their treatment course. One participant initiated treatment with right-sided inhibitory stimulation to HG, and left-sided inhibitory stimulation was added after the third session. The remaining participants received inhibitory stimulation to left HG.

### Data Analysis

Clinical and demographic variables, including presence or absence of depression, age, gender, type of rTMS stimulation, rTMS device, and baseline TFI score, were assessed for possible effects on treatment outcome. *T*-tests compared baseline characteristics for continuous variables; Fisher's Exact Method compared categorical variables. Repeated-measurement ANOVA analyzed both raw TFI score and percent change in TFI score over time. To determine if differences were present between subjects with and without co-morbid depression, this analysis also included group separation by presence of co-morbid depression diagnosis. Prior to repeated-measurement ANOVA with Greenhouse-Geisser correction, imputation of incomplete data (three subjects with one missing score each: one at session 5, two at session 10) was performed using a Markov chain Monte Carlo (MCMC) method with 5 iterations in SPSS v26.0 (selected via automatic method as described in SPSS support documentation) (47–50). Due to limitations of sample size, we did not perform a sensitivity analysis.

Primary outcomes included both raw and percent improvement in TFI score relative to baseline. Three time points were defined: session 1 (baseline), session 5, and session 10. Mean improvement and standard of deviation was calculated at each time point for the group of subjects with depression, the group without depression, and the pooled sample.

### Literature Review

Two authors (KGM and JCho) independently performed a systematic literature review in March 2021 to identify studies reporting the effects of sequential (i.e. combined frontal and temporoparietal) rTMS protocols for the treatment of tinnitus. Prior to the literature review, the authors defined and agreed upon the following inclusion criteria: English-language, reporting original data in human subjects, and including at least one study arm reporting the effects of multiple sessions of sequential rTMS treatment, without other interventions, on tinnitus. The “PubMed” database was searched with the keyword combination “(‘transcranial magnetic stimulation’ AND ‘tinnitus’) OR (‘TMS’ AND ‘tinnitus’).” In order to identify the maximum quantity of relevant reports, search terms were applied to all fields. Abstracts deemed potentially relevant upon initial review underwent full-text review by authors KGM and JCho. Reports meeting inclusion criteria had the following data abstracted for review: study design, number of subjects, presence or absence of comorbid MDD, total number of rTMS treatment sessions in the relevant study arm(s), rTMS treatment targets and localization methods in the relevant study arm(s), rTMS stimulation parameters in the relevant study arm(s), effect of rTMS treatment on outcomes of interest in the relevant study arm(s), response rates for sequential rTMS interventions, and any variables found to predict rTMS response. We used a modified version of the criteria applied by Lefaucheur et al. to grade the level of evidence for each included study, ranging from Class I (highest quality) to Class IV (lowest quality) (44) Class I describes randomized, sham-controlled trials with >25 participants receiving active treatment. Class II describes randomized, sham-controlled trials with <25 participants receiving active treatment (previous review of Lefaucheur et al. did not include studies with <10 participants; we included all studies with <25 participants); Class III describes other controlled studies of lower methodological quality with any number of participants; and Class IV describes uncontrolled studies, including case series and case reports. Findings of reviewed studies were reported, and common themes were elucidated and synthesized in a narrative format.

## Results

### Inclusion and Baseline Characteristics

Sample characteristics and rTMS parameters for included participants are presented in Table 1. There were no statistically significant differences in age, baseline TFI score, biological sex, type of rTMS machine, or type of rTMS stimulation between tinnitus patients with and without depression (Table 1).

### Tinnitus Changes Over the Course of Treatment/Outcomes

In terms of our analysis utilizing a repeated-measures ANOVA, after 5 independent imputations to address missing data, it demonstrated statistically significant improvement in both numeric TFI scores (corresponding F and p statistics given for each imputation: *F* = 10.77, *p* = 0.001; *F* = 7.41, *p* = 0.005, *F* = 8.31; *p* = 0.003, *F* = 9.92, *p* = 0.002; *F* = 6.60, *p* = 0.008) and percent improvement in TFI scores (corresponding F and p statistic pairs: *F* = 7.30, *p* = 0.006; *F* = 6.90, *p* = 0.007, *F* = 7.11; *p* = 0.006, *F* = 7.22, *p* = 0.006; *F* = 5.54, *p* = 0.015) over the course of treatment for the entire sample. All five independent imputations tested demonstrated a significant improvement within subjects over the course of treatment, though none demonstrated differences (or interaction effects related to the number of treatments) between those with and without co-morbid depression (all *F* < 2.0). The small sample size, in addition to the lack of between-group and group-by-session interactions in the ANOVA suggest that valid comparisons cannot be made within subgroups, although there may be differences in outcomes related to the presence or absence of depression and descriptive statistics may yield helpful insights.

In the aggregate pool of all subjects, subjects experienced an average 20% decrease in symptoms on the TFI after 10 treatments, with high inter-individual variability in outcome. One subject experienced greater than 50% reduction in symptoms, three experienced 25 to 50% reduction in symptoms, and five experienced less than 25% symptom reduction. In contrast, one experienced worsening symptoms (less than 25% symptom increase), which resolved within 2 weeks.

**Figure 1:**
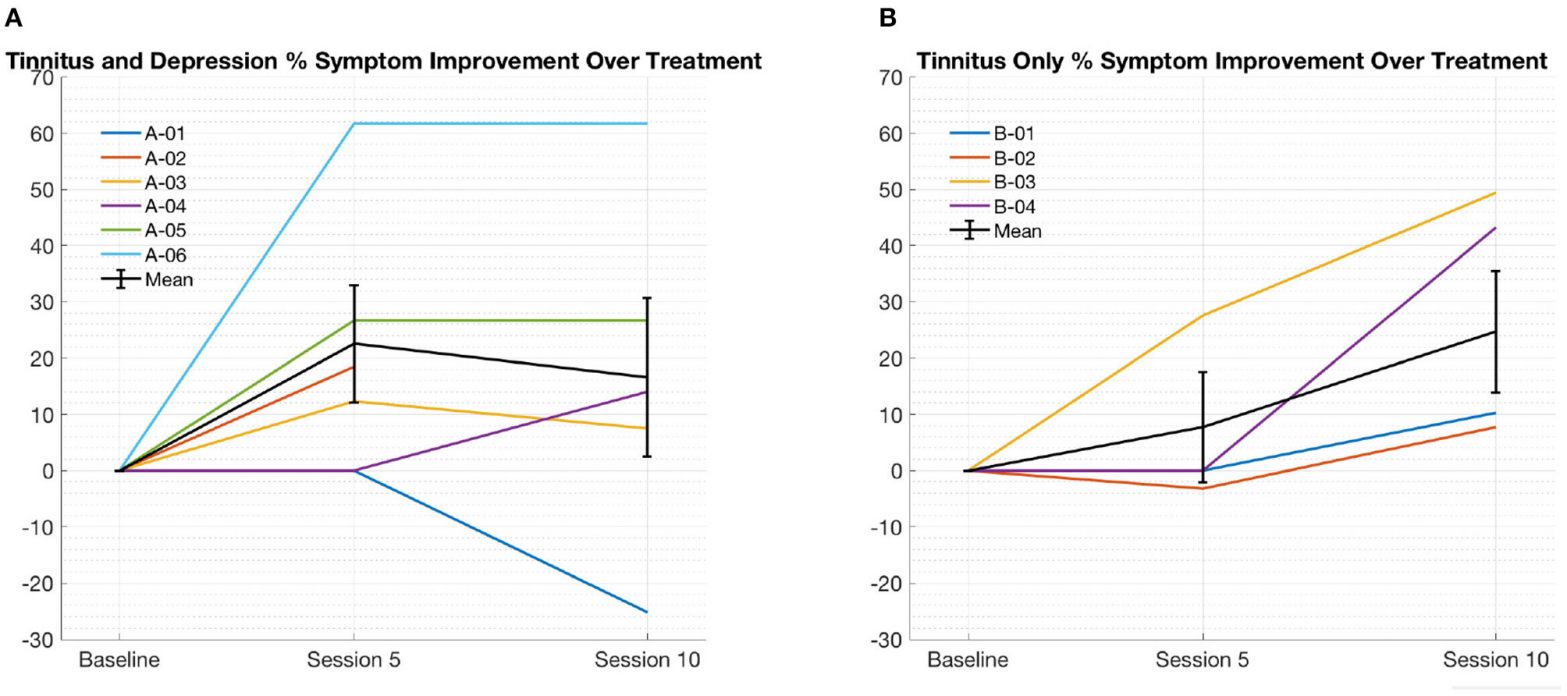
(**A**, depicted on the left) tinnitus and depression % improvement in TFI score from baseline over treatment. individual subjects and mean shown with standard error of the mean. (**B**, depicted on the right) tinnitus only % improvement in TFI Score from Baseline Over Treatment. Individual subjects and mean shown with Standard error of the mean.

Table 2 summarize the mean reduction in TFI score over the course of 10 sessions of rTMS treatment. Figure 1 shows improvement in TFI score severity over time for each subject by group. In subjects with tinnitus and depression (Figure 1A and Table 2), subjects experienced an average 17.1% decrease in symptoms on the TFI after 10 treatments, ranging from over 60% improvement to 25% worsening. Four of five subjects who reported symptom improvement at session 10 reported some benefit at treatment 5 as well (one did not have data available from treatment 5), though the magnitude of benefit varied from approximately 12% to over 60%. With respect to adverse events, one subject reported mild transient nausea and dizziness while receiving 10 Hz stimulation to left DLPFC; no other side effects were reported.

**Table 2:** Mean Tinnitus Functional Index (TFI) score percent change in each group over the course of 10 treatments.

	**Tinnitus only**		**Tinnitus and depression**		**Pooled aggregate**	
	***n =*** **4**		***n =*** **6**		***n =*** **10 (±SD)**	
**TFI Subsection**	**% Change by Tx 5**	**% Change by Tx 10**	**% Change by Tx 5**	**% Change by Tx 10**	**% Change by Tx 5**	**% Change by Tx 10**
Intrusiveness	4.2	31.3	17.1	16.4	12.3 (27.7)	23.0 (30.4)
Control	3.3	−8.9	18.4	3.1	12.7 (17.2)	−2.3 (32.3)
Cognition	5.2	38.3	25.8	18.6	18.1 (32.2)	27.4 (36.4)
Sleep	11.8	33.3	16.9	29.5	15.0 (20.0)	31.2 (30.2)
Hearing	19.9	33.5	22.9	21.0	21.9 (18.8)	25.7 (27.1)
Relaxation	−0.4	22.1	39.5	22.8	24.6 (34.6)	22.5 (34.5)
Quality of Life	13.4	35.1	31.3	14.1	24.6 (25.0)	23.4 (42.9)
Emotional	11.6	41.2	19.9	5.6	16.8 (23.9)	21.5 (40.6)
Total	8.1	27.7	23.8	17.0	18.0 (21.4)	21.7 (26.6)

*TFI measured at treatments five and ten. Percent change broken down by TFI subscale as well as total score. Positive percentage indicates improvement, negative indicates worsening*.

Subjects without comorbid MDD experienced an average 27.7% decrease in symptoms on the TFI after 10 treatments, again with high inter-individual variability in outcome, ranging from approximately 7 to 50% improvement (Figure 1B and Table 2). One of two subjects with > 40% symptom improvement after treatment 10 reported benefit by treatment 5 (the other had no treatment 5 data available). Both subjects with <40% improvement by session 10 reported no benefit at treatment 5. With respect to adverse events, one subject reported mild transient confusion and euphoria after treatment; no other side effects were reported.

### Results of Systematic Literature Review

**Figure 2:**
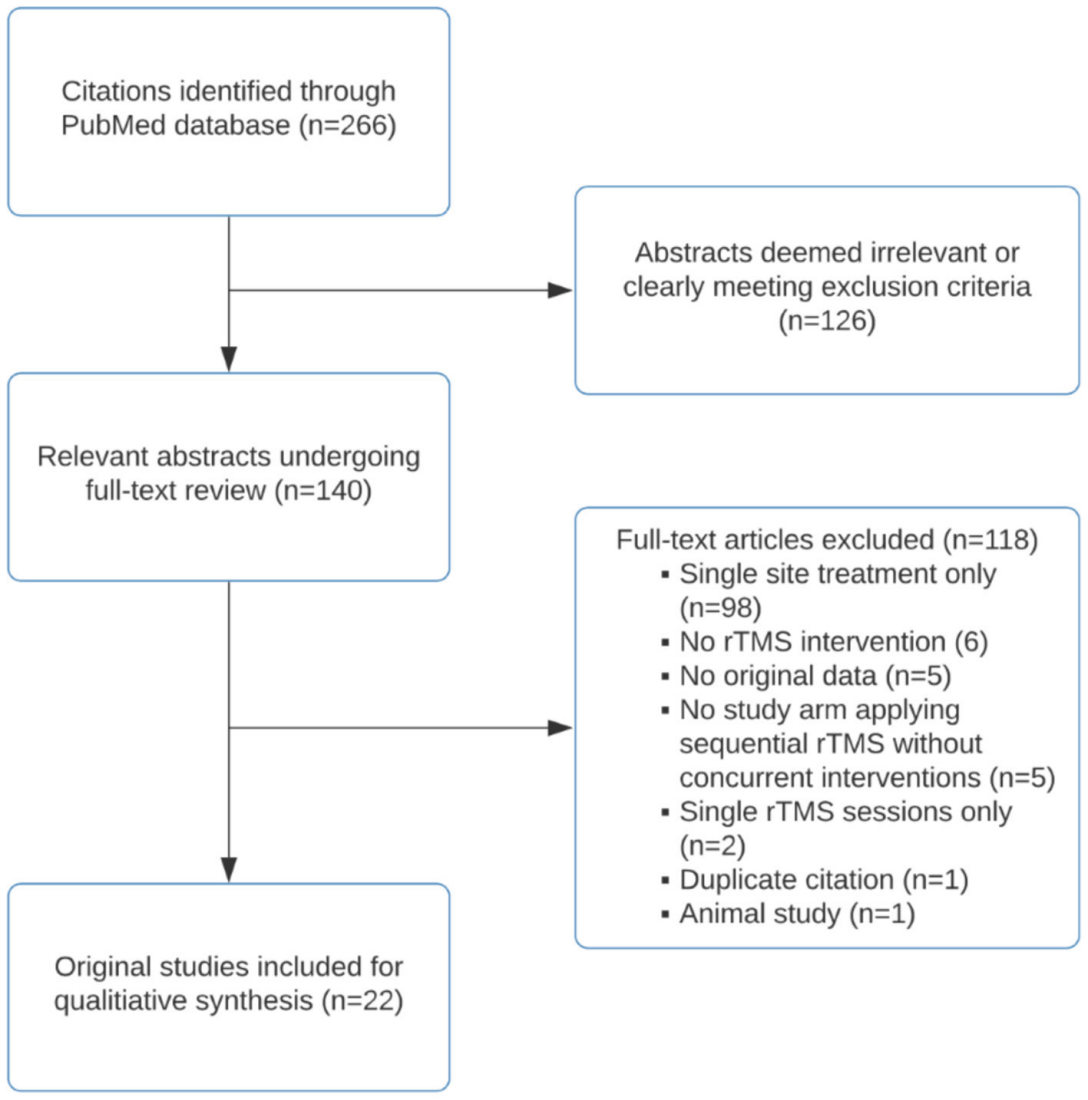
PRISMA flow diagram of literature review.

Using the strategy outlined in the methods section, search of the PubMed database resulted in 266 English language findings. After an initial screening for relevance, 126 citations were excluded. The remaining 140 citations underwent full-text review by authors KGM and JC. After full-text review, 118 studies were excluded due to the following: single site (including bilateral temporoparietal) stimulation only (*n* = 98), no rTMS intervention (*n* = 6), lack of original data (*n* = 5), sequential rTMS treatment arm involved other concurrent interventions (*n* = 5), single sessions of rTMS (*n* = 2), duplicate citations (*n* = 1), and animal study (*n* = 1). A total of 22 original studies were included for qualitative synthesis. For details of the screening and selection process, see the Preferred Reporting Items for Systematic Reviews and Meta-Analyses (PRISMA) Flow Diagram (Figure 2).

The methods and findings of the 22 included studies are summarized in Table 3 (17, 27, 35, 36, 38–43, 51–61). Methodology, including choice of rTMS stimulation parameters, was highly variable across studies. Eleven studies directly compared sequential rTMS approaches to single site approaches; of these, six demonstrated significant (*p* < 0.05) superiority of sequential rTMS for at least one outcome (17, 27, 35, 41, 52, 59). One favored sequential rTMS on a trend level (*p* < 0.1) (40) and the remaining four favored sequential rTMS on a descriptive level only (39, 51, 55, 58). Among four studies comparing sequential rTMS to sham rTMS, one demonstrated significant superiority of sequential rTMS (60) and the remaining three studies favored sequential rTMS on a trend level (36, 40, 42). Response rates for sequential rTMS protocols, where reported and with response criteria as defined by authors of each individual study, ranged from 26 to 92% (39, 41).

**Table 3:** Sequential prefrontal and temporo-parietal rTMS approaches in the treatment of tinnitus.

**References**	**Evidence grade**	**N**	**Targets**	**Localizing method**	**Parameters**	**Number of sessions**	**Comparison or control**	**Outcome**
Kleinjung et al. (17)	Class III	32 (16 in each group)	Single site: left AC. Sequential: left DLPFC, followed by left AC	Left DLPFC: 5 cm anterior to hand motor hotspot. Left AC: neuronavigation in both groups	Sequential: left DLPFC: 20 Hz, 1,000 pulses, 110% RMT, followed by L AC: 1 Hz, 1,000 pulses, 110% RMT	10	Single site: left AC, 1 Hz, 2,000 pulses, 110% RMT	Both groups improved, with no significant differences in TQ reduction between the groups immediately after treatment. At 90 day follow up, sequential group showed significantly greater improvement (*p =* 0.029). Sequential rTMS response rate: 50%
Burger et al. (39)	Class III	235 (single site: 188; sequential: 47)	Single site: left temporal cortex. Sequential: left DLPFC, followed by left temporal cortex	Pooled (10–20 system and neuronavigation)	Sequential: left DLPFC, 20 Hz, 1,000 pulses, 110% MT, followed by left temporal cortex: 1 Hz, 2,000 pulses, 110% RMT	10	Single site: left temporal cortex, 1 Hz, 2,000 pulses, 110% RMT	Higher response (>10 point reduction in TQ score) rates in sequential group (27.7%) than single site group (19.7%) on a descriptive level. Sequential rTMS response rate: 27.7%
Kreuzer et al. (51)	Class III	56 total (not specified by group)	Single site: left AC. Sequential: right DLPFC followed by left AC	Left DLPFC: 6 cm anterior to hand motor hotspot. L AC: not specified	Sequential: right DLPFC: 1 Hz, 1,000 pulses, 110% RMT, followed by left AC: 1 Hz, 2,000 pulses, 110% RMT	10	Single site: left auditory cortex: 1 Hz, 2,000 pulses, 110% RMT	No significant group differences, although sequential rTMS outperformed single site for all variables on a descriptive level with effect sizes ranging from 0.168 to 0.461. Sequential rTMS response rate: 40%
Lehner et al. (52)	Class III	538 (single site: 345; sequential: 193)	Single site: left AC. Sequential: left DLPFC followed by left AC	Pooled (neuronavigation and 10–20 system)	Sequential: left DLPFC: 20 Hz, 2,000 pulses, 110% RMT, followed by left AC: 1 Hz, 2,000 pulses, 110% RMT	10	Single site: left AC: 1 Hz, 2,000 pulses, 110% RMT	Significant decrease in TQ scores in both groups at day 12 with maintenance of significant decrease at day 90 in the sequential group only. Sequential rTMS response rate: 38%
Park et al. (41)	Class III (subjects' first course served as the control for their second course)	11 patients receiving 2 courses each (22 separate treatment courses)	Single site (initial course): AC (left, *n =* 8; right, *n =* 3). Sequential (second course): left DLPFC followed by left AC	Single site: neuronavigation for AC. Sequential: 10–20 system for right DLPFC (F4) and for AC (T3, T4)	Second course (1–6 months after 1^st^ course) administered sequential rTMS: right DLPFC: 1 Hz, 800 pulses, 110% RMT, followed by: T3 or T4: 1 Hz, 800 pulses, 110% RMT	10 (5 single site sessions, followed by 5 sequential sessions 1–6 months later)	Initial treatment course administered single site rTMS: AC (left = 8, right = 3): 1 Hz, 800 pulses, 110% RMT	The second (sequential) rTMS course led to signific antly greater reductions in THI and VAS scores than did the first (single site) course (*p <* 0.05). Sequential rTMS response rate: 73%
Lehner et al. (27)	Class III	74 (single site: 29; sequential: 45)	Single site (historical control): left AC. Sequential: left DLPFC, followed by left TPJ, then right TPJ	Single site: 10–20 system (Langguth 2,006 method) for left AC. Sequential: 6 cm anterior to hand motor hotspot for left DLPFC; 10–20 system (midway between T3-P3 and T4-P4) for TPJ	Sequential: left DLPFC: 20 Hz, 2,000 pulses, 110% RMT, followed by left, then right TPJ: 1 Hz, 2,000 pulses each, 110% RMT	10	Single site (historical control): left AC: 1 Hz, 2,000 pulses, 110% RMT	Both groups improved, with no significant differences between groups immediately after treatment. At day 90, significantly greater improvement in TQ scores in the sequential group (*p =* 0.045). Sequential rTMS response rate: 49%
Langguth et al. (42)	Class I	188 (single site, 10–20 localized: 48; single site, neuro-navigated: 48; sham rTMS: 45; sequential rTMS: 47)	Single site: left AC. Sequential: left DLPFC then AC	Pooled (10–20 system and neuronavigation)	Sequential: left DLPFC: 20 Hz, 2,000 pulses, 110% RMT, followed by left AC: 1 Hz, 2,000 pulses, 110% RMT	10	Single site group: left AC: 1 Hz, 2,000 pulses, 110% RMT, and sham rTMS group	No significant time by group effect, but in an exploratory analysis, sequential group showed greater change in TQ score compared to sham (Cohen's d=0.405) on a trend level (*p =* 0.056). Sequential rTMS response rate: 43%
Cristancho et al. (53)	Class IV	5	Left DLPFC, followed by left TPC	Left DLPFC: Beam F3 method. TPC: 10–20 system (midway between C3-T5).	Left DLPFC: 10 Hz, 4,000 pulses, 110% RMT, followed by TPC: 1 Hz, 1,800 pulses, 110% RMT	10	None	Mean change in THI score was 12 points. Sequential rTMS response rate: 60%
Park et al. (54)	Class III	14 (6 in group 1; 8 in group 2)	Left AC, followed by left DLPFC in both groups	10–20 system for both AC and DLPFC	Both groups received sequential rTMS with varying pulse numbers. Group 1: left AC: 1 Hz, 1,000 pulses, 110% MT, followed by left DLPFC: 1 Hz, 1,000 pulses, 110% MT)	Group 1: 3; group 2: 4	Group 2 (also received sequential rTMS): left AC: 1 Hz, 2,000 pulses, 110% MT, followed by left DLPFC: 1 Hz, 1,000 pulses, 110% MT	Group 1 showed no significant reduction in THI score. At 2 weeks, group 2 showed a significantly greater THI reduction than group 1 (*p =* 0.028).
Lehner et al. (55)	Class IV	55 patients each completing two distinct courses (110 rTMS courses with 6 different protocols)	Protocols 1 and 2: targeted AC only. Protocols 3 and 4: left DLPFC, followed by AC. Protocol 5: targeted left DLPFC, followed by bilateral TPC. Protocol 6: medial frontal cortex, followed by left TPC	Pooled (neuronavigation and 10–20 system)	Single site protocols: left AC: 1 Hz, 2,000 pulses vs. 4,000 pulses total, 110% RMT. Sequential protocols: 20 Hz at left DLPFC followed by 1 Hz at left AC, 2,000 pulses vs. 4,000 pulses total, 110% RMT; 20 Hz at left DLPFC followed by 1 Hz at bilateral TPC, 4,000 pulses total, 110% RMT; 10 Hz at medial frontal cortex with double cone coil at 110% RMT followed by 1 Hz at left TPC at 110% RMT, 4,000 pulses total	10 (for all protocols)	None	Both first and second courses significantly reduced tinnitus severity by TQ score (*p =* 0.002 and *p* < 0.001 respectively). No protocol was significantly superior to the others. On a descriptive level, a sequential protocol with 20 Hz at left DLPFC followed by 1 Hz at bilateral TPC led to largest TQ reductions in both first and second courses.
Kreuzer et al. (43)	Class III	36 (18 each)	Comparison of two sequential protocols, with either medial frontal rTMS (arm 1) or left DLPFC rTMS (arm 2), followed by left TPC stimulation in both arms	Arm 1: medial PFC: 10-20 system (1.5 cm anterior to 1/3^rd^ of the distance from nasion to inion). TPC: 10-20 system (midpoint of C3-T5). Arm 2: left DLPFC: 6 cm anterior to hand motor hotspot. TPC: 10–20 system (midpoint of C3-T5)	Arm 1: medial frontal stimulation with double cone coil: 10 Hz, 2,000 pulses, 100% RMT, followed by left TPC with figure-of-eight coil: 1 Hz, 2,000 pulses, 110% RMT	10	Arm 2 (also sequential rTMS): left DLPFC: 10 Hz, 2,000 pulses, 110% RMT, followed by left TPC: 1 Hz, 2,000 pulses, 110% RMT	Significant reductions in TQ score in both groups but with no time by group interaction and no differences in response rates between arms. Sequential rTMS response rate: ranged 28-33%
Lehner et al. (40)	Class II	49 (single site: 24; sequential: 25; historical control group: 25)	Single site: left TPJ. Sequential: left DLPFC, followed by bilateral TPJ.	Left DLPFC: 6 cm anterior to hand motor hotspot. TPJ: 10–20 system (midpoint between T3-P3 or T4-P4)	Sequential: left DLPFC: 20 Hz, 1,000 pulses, 110% RMT, followed by left and then right TPJ, each with: 1 Hz, 1,000 pulses, 110% RMT	10	Single site rTMS: left TPJ, 1 Hz, 3,000 pulses, 110% RMT, and historical control with sham RTMS	Both sequential and single site groups showed significant reductions in TQ score at day 12 (*p* < 0.001) and both were superior to placebo, but with no difference between the two active groups. The sequential group showed numerically larger reductions in TQ score at day 90 compared to sham and single site (likely significant but *p* values not reported) and day 180 compared to the single site group (authors report trend level; p value not reported). Sequential rTMS response rate: 40% (day 12) and 52% (day 90)
Kreuzer et al. (38)	Class III	24 (individual protocol: 12; standard protocol: 12)	Individualized group: either left or right DLPFC, followed by either left or right temporo-parietal junction (TPJ). Standard group: left DLPFC, followed by either left or bilateral TPJ	Left DLPFC: 6 cm anterior to hand motor hotspot. TPJ: 10–20 system (midway between T3-P3 or T4-P4)	Two groups received sequential rTMS. Patients with immediate tinnitus reduction during a test session (12/25) received individualized treatment, while those with no response received a standard sequential rTMS protocol. Individualized group: Prefrontal: 9/12 left DLPFC and 3/12 right; 5 Hz (*n =* 2), 10 Hz (*n =* 1), 20 Hz (*n =* 6), cTBS (*n =* 3). TPJ: 7/12 left and 5/12 right; 5 Hz (*n =* 3), 10 Hz (*n =* 3), 20 Hz (*n =* 2), cTBS (*n =* 4). All at 110% RMT	10	Standard group: rTMS to left DLPFC: 20 Hz, 2,000 pulses, 110% RMT, followed by either left TPJ stimulation (*n =* 3), 1 Hz, 2,000 pulses, 110% RMT, or bilateral TPJ stimulation (*n =* 9), 1 Hz, 1,000 pulses each side, 110% RMT	Individual treatment led to non-significantly greater reductions in TQ scores compared to standard treatment, with moderate-to-large effect sizes (0.465 at 2 weeks, 0.816 at 12 weeks). Individualized treatment led to non-significantly higher response rates (58% at 2 weeks, 67% at 12 weeks) than did standard treatment (42% at both 2 and 12 weeks). Sequential rTMS response rates ranged 42-67%
Noh et al. (35)	Class III	17 (9 sequential, 8 single site)	Single site: left DLPFC. Sequential: left DLPFC and left AC (order of stimulation not specified)	Left AC: 10–20 system (Langguth 2,006 method). Left DLPFC: 10-20 system (F3)	Sequential: left DLPFC: 1 Hz, 1,000 pulses, 110% RMT, and left AC: 1 Hz, 2000 pulses, 110% RMT (order of stimulation not specified)	4	Single site: left DLPFC: 1 Hz, 3,000 pulses, 110% RMT	Sequential group showed significantly greater improvement than single site group at all time points (1 week, *p <* 0.001, 2 weeks, *p <* 0.001, 4 weeks, *p <* 0.001, and 12 weeks, *p* = 0.002). Sequential rTMS response rate: 89%
Noh et al. (35)	Class IV	22 (10–20 group: 9; neuronavigation group: 13)	Both groups: left DLPFC, followed by left AC	10–20 group: 10–20 system used to localize DLPFC and AC. Neuronavigation group: 10–20 system used for DLPFC and neuronavigation for AC	Both groups treated with same parameters: Left DLPFC: 1 Hz, 1,000 pulses, 110% RMT, followed by left AC: 1 Hz, 2,000 pulses, 110% RMT	4	Neuronavigation versus 10–20 system	Both groups had significant improvement in THI score. No significant difference between groups. Sequential rTMS response rates: 89-92%
Poeppl et al. (56)	Class IV	60	Left DLPFC, followed by left AC	Left DLPFC: 6 cm anterior of hand motor hotspot. Left AC: 10–20 system (Langguth 2006 method)	Sequential: left DLPFC: 20 Hz, 2,000 pulses, 110% RMT, followed by left AC: 1 Hz, 2,000 pulses, 110% RMT	10	None	Sequential rTMS response rate: 37%. On structural MRI, responders demonstrated changes in left DLPFC, left operculo-insular, and right inferior temporal cortex gray matter, while non-responders did not.
Formánek et al. (36)	Class II	53 (rTMS: 20; sham: 12; ginkgo biloba: 21)	Left DLPFC and bilateral AC	Neuronavigation	Left DLPFC: 25 Hz, 300 pulses, 80% RMT, followed by bilateral AC: 1 Hz, 1000 pulses, 110% RMT	5	Sham rTMS or ginkgo biloba extract	No significant effect of rTMS compared to sham or ginkgo biloba for THQ or TRQ scores at 1 and 6 months. Small/clinically irrelevant trend toward greater improvement in THI score with sequential rTMS at 1 and 6 months.
Kar et al. (57)	Class IV	1	First 5 sessions: left DLPFC followed by left TPJ. Last 5 sessions: left DLPFC followed by right TPJ	Left DLPFC: 5 cm anterior to hand motor hotspot. TPJ: 10–20 system (left TPJ: midpoint of T3-P3; right TPJ: midpoint of T4-P4).	All sessions included: left DLPFC: 10 Hz, 600 pulses, 110% RMT, followed by left (first 5 sessions) or right (last 5 sessions) TPJ: 1 Hz, 1,200 pulses, 110% MT	10	None	On a Likert scale, the patient demonstrated a 39% reduction in tinnitus symptoms at the end of treatment and a 44% reduction at 3 week follow up.
Kyong et al. (58)	Class II	24 (sequential: 8; single site: 8; sham: 8)	Single site: left AC. Sequential: left DLPFC, followed by left AC	10–20 system for all targets (left DLPFC: F3; left AC: T3)	Single site: Left AC: 1 Hz, 3,000 pulses, 110% RMT. Sequential: left DLPFC: 1 Hz, 1,000 pulses, 110% RMT, followed by left AC: 1 Hz, 2,000 pulses, 110% RMT	4	Sham (tilted coil at T3)	Sequential group had greater reductions in THI than the single site or sham groups on a descriptive level. There were higher response rates in the sequential group (5/8) than single site group (3/8). The sequential group demonstrated significantly larger changes in cortical inhibition compared to sham (*p =* 0.024) or single site (*p =* 0.023) groups, and changes in cortical inhibition were correlated with changes in THI (*p =* 0.04). Sequential rTMS response rate: 62.5%
Noh et al. (59)	Class II	17 (sequential: 9, single site: 8)	Single site: left AC. Sequential: left DLPFC, followed by left AC	Left AC: 10–20 system (Langguth 2006 method). DLPFC: 10-20 system (F3).	Sequential: left DLPFC: 1 Hz, 1,000 pulses, 110% RMT, followed by left AC: 1 Hz, 2000 pulses, 110% RMT	4	Single site: left DLPFC, 1 Hz, 3,000 pulses, 110% RMT	Sequential group demonstrated significantly greater reductions in THI score compared to single site group at 1 week (*p <* 0.001), 2 weeks (*p <* 0.001), 4 weeks (*p <* 0.001), and 12 weeks (*p* = 0.002) after rTMS treatment. Sequential rTMS response rate: 88.9% (compared to 37.4% in single site group).
Noh et al. (60)	Class II	48 (sequential: 16; single site: 16; sham: 16)	Single site: left AC. Sequential: left DLPFC and left AC.	Left DLPFC: 10–20 system (F3). Left AC: 10–20 system (Langguth 2006 method).	Sequential: Left DLPFC: 1 Hz, 1,000 pulses, 110% RMT, followed by left AC: 1 Hz, 2,000 pulses, 110% RMT	4	Single site: left AC: 1 Hz, 3,000 pulses, 110% RMT. Sham: tilted coil at LDLPFC and left AC	Sequential group demonstrated significant reduction in THI and VAS scores at 4 (*p* = 0.011), 8 (*p* = 0.03), and 12 (*p* = 0.014) weeks, while single site and sham groups did not. The average THI reduction was significantly greater at 4 weeks for sequential group compared to sham (*p =* 0.015). Sequential rTMS response rate: 62.5%
Kim et al. (61)	Class IV	10 (and 10 age-matched healthy controls)	Left DLPFC and left AC	Left DLPFC: 10–20 system (F3). Left AC: neuronavigation	Left AC: 1 Hz, 2,000 pulses, 110% RMT, followed by left DLPFC: 1 Hz, 1,000 pulses, 110% RMT	4	Healthy controls also underwent TMS with same parameters	Tinnitus patients experienced average THI reduction of 16.9 points; THI response correlated with changes in functional connectivity of left auditory cortex but not DLPFC. Sequential rTMS response rate: 90%

*AC, primary auditory cortex; DLPFC, Dorsolateral prefrontal cortex; RMT, Resting motor threshold; THI, Tinnitus Handicap Inventory; THQ, Tinnitus Handicap Questionnaire; TPC, Temporoparietal cortex; TPJ, temporoparietal junction; TQ, Tinnitus Questionnaire; TRQ, Tinnitus Reaction Questionnaire*.

Only one study explicitly included currently depressed subjects; in this study, eight of eleven subjects were depressed (Beck Depression Inventory scores ranging 11–36) (41). There was no significant improvement in depressive symptoms with sequential rTMS (41). Tinnitus response rates were not significantly different between subjects with depression (75%) and those without depression (67%) (41). However, presence of mild depression was associated with greater tinnitus improvement after sequential rTMS than was the absence of depression or the presence of moderate-to-severe depression (41). Whereas two studies reported that baseline depression severity (BDI) score did not predict rTMS outcome for tinnitus (39, 51), another study showed that baseline depression severity (BDI) score predicted tinnitus response at day 12, but only for single site stimulation (52).

Several studies examined predictors of tinnitus response to rTMS. Higher baseline tinnitus severity (TQ score) predicted favorable response in two studies (39, 52) but failed to predict outcome in two other studies (51, 56). Worsening of THI and TQ scores between screening and pre-treatment baseline predicted favorable response to rTMS in one study (51) and, in another study, patients whose tinnitus severity improved between screening to pre-treatment baseline benefited less than patients without initial improvement (52). Similarly, in a study examining response across two separate courses of rTMS for tinnitus, worsening of the TQ score in the inter-treatment interval better predicted outcome of the second treatment course than did response to the first treatment course (55).

In one study that employed individualized treatment parameters in comparison to a standardized sequential rTMS protocol, response to a single, test treatment session predicted the effect of daily rTMS treatment (38). This study also demonstrated a significant correlation between right-handedness and benefit from left frontal stimulation (38). In another study, the presence of temporomandibular complaints predicted favorable tinnitus response to sequential rTMS (52).

In three studies, tinnitus duration and presence of hearing loss failed to predict rTMS outcome (36, 51, 56). Gender, age, and tinnitus laterality did not predict rTMS outcome in two studies (51, 56). In another study, age and mean hearing loss were only weakly correlated with TQ changes (42). Therapeutic outcome of rTMS was not predicted by intermittent versus continuous experience of tinnitus (52), number of previous treatment trials (51), education level (36), or baseline brain morphology (56).

Sequential rTMS protocols typically targeted left DLPFC and either left primary auditory cortex or left temporoparietal cortex. Left auditory cortex was usually stimulated with 1 Hz treatment. Frontal stimulation parameters varied significantly between groups, with one group (38) tending to employ high frequency (20 Hz) treatment at left DLPFC, and another group (60) tending to study low frequency (1 Hz) at left DLPFC. In general, sequential treatment protocols administering 1 Hz at left DLPFC were superior to single site stimulation in the short-term (1–12 weeks) (35, 58–60), while sequential treatment protocols administering 20 Hz at left DLPFC were superior to single site stimulation primarily in the longer term (90–180 days) (17, 27, 40, 52).

## Discussion

To date, rTMS is not a first line treatment for tinnitus, as the literature describing its efficacy reports mixed results (2, 44). A 2019 systematic review of rTMS for tinnitus treatment included 39 study arms, of which 31 reported outcomes; based upon these results, use of rTMS was not recommended (2, 31). Similarly, a recent meta-analysis found no significant effect of low frequency tinnitus treatment when compared to sham, although nine out of the ten included studies employed single site approaches (62). However, another recent meta-analysis found that rTMS for tinnitus was superior to sham, and successfully reduced tinnitus symptoms for 1 week to 6 months after intervention (63). Additionally, another recent meta-analysis of randomized controlled trials found no significant benefit immediately following treatment, but found significant, beneficial effects of rTMS compared to sham at 1 and 6 months (64). Finally, a recent meta-analysis reported that sequential rTMS approaches were more efficacious (SMD −0.72 to −0.57) than single site approaches (SMD −0.3 to −0.45), with the exception of bilateral cTBS at temporal cortices (SMD −0.79) (65). The same study reported that stimulation of the frontal cortex was the second most effective rTMS approach for tinnitus (SMD −0.94) (65).

The profound heterogeneity of treatment protocols employed across studies of rTMS for treatment of tinnitus may explain the dramatically different conclusions reached by these recent systematic reviews and meta-analyses. For example, Dong et al. primarily included single site approaches, and reported no significant effects of rTMS, while Chen et al. included sequential approaches and found a significant effect of rTMS, with large effect sizes (62, 65). The heterogeneity of treatment protocols studied thus far makes it difficult to determine which rTMS protocol is most effective for the treatment of tinnitus, although the findings of Chen et al. suggest that sequential rTMS approaches—as used in the present case series—are more effective than single site strategies (65). Still, it remains to be determined what target(s) or combination of targets, and which pattern(s) of stimulation are optimal. Auditory cortical targets are typically treated with inhibitory (i.e. 1 Hz or cTBS) protocols, although a recent retrospective case series applied a novel stimulation protocol called Alpha Burst Stimulation to auditory cortex, with promising results (66). Frontal targets have been stimulated with both inhibitory and excitatory protocols, with mixed results; according to our literature review, the application of 1 Hz treatment at left DLPFC demonstrated greater benefit than single site stimulation in the short-term, while the application of 20 Hz treatment at left DLPFC demonstrated greater benefit than single site stimulation in the long-term. In the present case series, a sequential rTMS approach involving excitatory treatment at left DLPFC and inhibitory treatment at auditory cortex led to an average 20% reduction in tinnitus symptoms.

The DLPFC is part of the temporo-prefrontal network, which is considered critical for transient storage of auditory stimuli (67). It is plausible that inhibitory (e.g. 1 Hz) treatment at DLPFC reduces the tinnitus percept by disrupting its storage or maintenance within this network (41). On the other hand, the limbic system has been postulated to act as a descending “noise-cancellation mechanism” in tinnitus, in which case excitatory, (e.g. 10 or 20 Hz) treatment at DLPFC may aid in suppressing the perception of tinnitus (23). Tinnitus distress has also been attributed to “hyperactive attention,” in which case stimulation of the DLPFC may reduce tinnitus distress by improving attentional control (68). Ultimately, the neurobiology of tinnitus has yet to be fully elucidated. While non-auditory brain regions—the so-called “tinnitus distress network”—are clearly implicated in the pathophysiology tinnitus, the precise role of attentional/limbic networks in tinnitus pathophysiology remains unclear, as does the optimal manner of engaging these networks. With this lack of clarity, it is particularly interesting to examine the association of pulse number and stimulation intensity with symptom improvement. As Schoisswohl et al. found, lower stimulation intensity and lower pulse number were more likely to be associated with positive outcomes, though the reason for this remains unclear (31).

Depression and tinnitus are commonly comorbid, and rTMS would seem uniquely suited to treat both conditions simultaneously. However, the effect of comorbid depression on the outcome of rTMS treatment of tinnitus remains unclear (39, 51, 52). In some studies, severity of depression did not predict tinnitus response to rTMS (39, 51), while another study found that individuals with tinnitus and mild depression exhibited greater tinnitus improvement after sequential rTMS than subjects with either no depression or moderate-to-severe depression (41). In the present case series, tinnitus patients without depression had a greater average reduction in tinnitus symptoms than did patients with comorbid depression. Further work is needed to clarify the effects of comorbid depression on tinnitus response to rTMS—and what this may indicate about the neurobiology of tinnitus. On the other hand, it seems clear that rTMS treatment of tinnitus—even sequential approaches engaging DLPFC—does not improve depressive symptoms. This is most likely due to the low number of treatment sessions employed in trials of rTMS for tinnitus.

In the present case series, nine of the ten subjects experienced at least some improvement in tinnitus symptoms after rTMS, but there was marked heterogeneity in treatment outcomes across participants. While this finding may be an artifact of our sample size, it may also be attributable to heterogeneity in the neurophysiology of tinnitus in different individuals. We observed a potential bimodal response to rTMS, in which subjects with tinnitus only tended to improve either only slightly (5–10%) or substantially (>40%) (Figure 1B, Table 2). This finding in our small sample, though preliminary at best, may indicate that rTMS stimulation engages the selected target(s) in some, but not all, subjects. “Precision TMS” approaches use neurophysiology or neuroimaging to guide target selection in an attempt to address this issue. However, the evidence to date suggests that these approaches are of limited utility in tinnitus treatment (31, 69). Another plausible explanation for this possible bimodal response is that the neurophysiology of tinnitus is highly individual in nature. Treating patients with a standard protocol, as in this case series and much of the literature to date, may only adequately address the pathophysiology of tinnitus in a subset of patients. Individualized treatment approaches, in which patient's response to a single session guides selection of parameters for the entire course of treatment, have been employed, with promising results (38).

The present case series has several key limitations. Our most pronounced limitation is the small sample size (*n* = 10), which may underlie the heterogeneity observed in rTMS response and lack of conclusive analyses. Due to the lack of sham control in this study, we cannot determine whether the observed improvement was a treatment effect (i.e. alteration of neuronal excitability in limbic and auditory networks), natural variability in symptoms, or placebo response. The heterogeneity in stimulation parameters in the case series, with different subjects receiving different stimulation protocols, is another limitation of our study.

The evidence supporting use of sequential rTMS approaches in tinnitus is increasingly positive, with multiple meta-analyses supporting efficacy in the short and long term. Still, there is much to be clarified, and much room for improvement in outcomes. Our literature review suggested that 1 Hz treatment at left DLPFC is particularly promising. Future studies should directly compare 1 Hz treatment at left DLPFC with high-frequency treatments, including 20 Hz stimulation, as well as 10 Hz and iTBS, which were employed in the present case series with promising results. Future studies should also more closely examine the optimal treatment intensity and pulse number, including lower stimulation intensities and pulse numbers. As prior work has suggested, it may be that “less is more” and there is a therapeutic window for these treatment parameters (31). Individualized treatment parameters based on response to a single, test session are another promising approach that merit further study. However, not all patients respond to a single, test session. Future studies might also attempt to identify predictors of rTMS response for tinnitus, and seek to identify whether any demographic or clinical data might be used to guide selection of the optimal treatment protocol. There is a pressing need for safe and effective tinnitus treatment, and with further study, sequential rTMS approaches may be able to fill this need.

## Data Availability Statement

The raw data supporting the conclusions of this article will be made available by the authors, without undue reservation.

## Ethics Statement

The studies involving human participants were reviewed and approved by the Medical Institutional Review Board (IRB) of the University of California, Los Angeles (UCLA) Office of the Human Research Protection Program. The patients/participants provided their written informed consent to participate in this study. Written informed consent was obtained from the patients/participants for the publication of any potentially identifiable images or data included in this article.

## Author Contributions

KM, DK, NG, JCL, SW, RT, JL, and AL designed treatment and study protocol and performed TMS treatments for the study. RC, AW, JCo, and ML gathered and analyzed case series data with additional input from AL. All authors contributed to data interpretation. JCh and KM primarily performed the literature review with additional input from AL, AI, and ML. KM, JCh, RC, and ML primarily drafted the paper, with additional input from AI and AL. All authors assisted in manuscript editing and approved the final manuscript.

## Funding

This project was made possible by the Ryan Family Fund for TMS Research.

## Conflict of Interest

AW has served as a consultant to HeartCloud, Inc. within the past 36 months. JCL has received equipment in-kind support from Magventure Inc. AL discloses that within the past 36 months he has received research support from the National Institutes of Health, Department of Defense, and NeuroSigma, Inc. He has served as a consultant to NeoSync, Inc., and ElMindA. He is Chief Scientific Officer of Brain Biomarker Analytics LLC (BBA). ML has equity interest in BBA. ML discloses he has served as a consultant to Neuroelectrics, Inc. within the past 36 months. The remaining authors declare that the research was conducted in the absence of any commercial or financial relationships that could be construed as a potential conflict of interest.

## Publisher's Note

All claims expressed in this article are solely those of the authors and do not necessarily represent those of their affiliated organizations, or those of the publisher, the editors and the reviewers. Any product that may be evaluated in this article, or claim that may be made by its manufacturer, is not guaranteed or endorsed by the publisher.
